# Biosensors with Built-In Biomolecular Logic Gates for Practical Applications

**DOI:** 10.3390/bios4030273

**Published:** 2014-08-27

**Authors:** Yu-Hsuan Lai, Sin-Cih Sun, Min-Chieh Chuang

**Affiliations:** Department of Chemistry, Tunghai University, Taichung 40704, Taiwan; E-Mails: karenlai0222@gmail.com (Y.-H.L.); sandy79325@gmail.com (S.-C.S.)

**Keywords:** biosensor, molecular computing, logic gate, biomedical application

## Abstract

Molecular logic gates, designs constructed with biological and chemical molecules, have emerged as an alternative computing approach to silicon-based logic operations. These molecular computers are capable of receiving and integrating multiple stimuli of biochemical significance to generate a definitive output, opening a new research avenue to advanced diagnostics and therapeutics which demand handling of complex factors and precise control. In molecularly gated devices, Boolean logic computations can be activated by specific inputs and accurately processed via bio-recognition, bio-catalysis, and selective chemical reactions. In this review, we survey recent advances of the molecular logic approaches to practical applications of biosensors, including designs constructed with proteins, enzymes, nucleic acids, nanomaterials, and organic compounds, as well as the research avenues for future development of digitally operating “sense and act” schemes that logically process biochemical signals through networked circuits to implement intelligent control systems.

## 1. Introduction

A biosensor, a device that responds to a particular analyte in a selective way, incorporates “biological recognition” as sensing elements connected to a “transducer”. Recently, a number of innovative works have demonstrated the feasible embodiment of integrating biological recognition as well as a transducer into a digital utility by configuring versatile tailor-designed biochemical reactions. Particularly distinct from conventional characteristics of biosensors, the implemented integration is capable of handling multiple analytes simultaneously (different from the determination of a “single analyte” in every sensing/sensor) to give a digital output (*i.e*., a qualitative YES/NO answer, which is contrary to quantitative measurements in the commonly referred sensors). Conceptually, while the functional body of logic designs substantially resembles biosensors in an “array” fashion, such digitally built-in sensing devices are endowed with the unique ability to chemically interpret a conclusion from the measurement/sensing, computed via the integrated molecular reactions, which mimic Boolean logic gates.

Boolean logic gates are physical devices that exert logical operations by receiving dual or multiple inputs and yielding a single digital output. This principle of logical operations is the fundamental core of widespread silicon-based computing. Analogously, molecular computing processes chemical information via cascades of biocatalytic or bio-affinity events to generate an observed change of a physical signal as an output. In particular, the biocatalytic or bio-affinity activity relies on specific interactions between the employed molecules and its ligand. For instance, the biocatalytic process, mainly executed by enzymes, is to expedite the chemical transformation of specific (or a limited group of) substrates. Hybridization of complementary DNA is another example; this event exhibits high specificity conferred by hydrogen bonds formed between specific bases. For these events, any combination except for the preferred coupling is unfavorable. This inherent nature given by these biochemical activities uniquely emulates the logics of Boolean **AND** and **OR** gates. Therefore, biocatalytic or bio-affinity activity can be the central constituent to benefit from a configuration of a biomolecular logical system. Moreover, cascades of such biochemical events enable functionalities of logical operations and allow the (bio-)chemical output displayed by optical and electrochemical transducers.

Seeing the tremendously complex genetic codes of programming algorithms in biology, genetic determination of a single target is insufficient to vividly describe biological systems. For instance, expression patterns of miRNAs associated with cancers and other dysfunctions often involve simultaneous up (or down) regulation of multiple miRNAs [1]. These features underscore the urgent significance of concurrent analysis of multiple targets of interest and manifest the integrity to develop the logical gates-based biosensors. Boolean logical gates, on the one hand, perform computations induced by dual or multiple inputs; and by virtue of this feature, the logical gates built-in biosensors hold the inherent capability required to analyze multi-analytes. On the other hand, the logical operation ideally rapidly generates a digital output represented by **0** and **1** upon receiving the input information, which doubtlessly meets the crucial requirement to offer an immediate response leading to an exceptional high-fidelity “fast-screening” diagnostic tool. The uniqueness inspires the emerging exploitation of logical gate-based biosensors and confers expectable success on realization of ultimate biosensing applications.

This review gives a summary of recent development of biomolecular logical gate-based biosensors. We present a variety of processing strategies for sensing purposes, which are categorized in accordance with their core element used to construct the molecular recognition and transduction utilities, along with significant advances in connection with the actuation and treatment. Albeit enzyme- [2,3] and DNA-based [4,5] logic built-in biosensing technologies have been reviewed separately in the past, this review aims at filling the gap by considering the merits in practical usage. Furthermore, we underline the exploitation which integrated multiple modalities into a single device. Since the principal interest of the present review addresses the endeavors which explore the extreme applications of sensors with built-in biomolecular logical gates, the studies which indicated the specific application scope and evaluate diverse circumstances that potentially occur in association with the specified application, are summarized, yet proof-of-concept achievements which implemented biomolecular logical gates are excluded.

## 2. Recognition and Transduction Strategies of Logically Gated Sensing Activity

Unexceptional to the essential components involved in conventional sensors, the logical gate built-in biosensors are composed of three fundamental elements: (1) an analyte which is inputted into the biomolecular processing of the system; the inputs could be proteins, ions, nucleic acids, or small compounds; (2) a recognition mechanism which is able to react specifically with the inputted signal, programmed by the well-designed biochemical activity. When receiving the correct analyte, the recognition event ultimately transforms the input to a measurable output; (3) the output which is transduced and presented in a physical or chemical format quantitatively or qualitatively reporting the state of input. Distinct from the concept commonly given by traditional biosensors, the logical gate based biosensors exhibits the extraordinary characteristics including: (1) multiple analytes are inputted concurrently; (2) rather complex and concatenated biochemical reactions are programmed to process the multiple inputs in accordance with the built-in Boolean logic; (3) a digital output is generated to interpret the result of the multiplex analysis in a “YES/NO” format.

It is noted that the biomolecular recognition is generally equipped with a cascade of bioaffinity or biocatalytic events to implement analogous functionality of logical gate programming. Such cascaded biochemical events can be constructed based on integrated enzymatic reactions in parallel or series. Bioaffinity reactions, meanwhile, are manipulated on the basis of specific reactions between nucleic acids, bioreceptors, and ligands, or antibodies and antigens. Alternatively, oligonucleotides such as aptamers or DNAzymes, endowed with favorable functional properties, vastly expand the scope of inputs to non-nucleic acids entities such as proteins, ions, and small molecules in the DNA-based reactions. This versatility substantially enhances the complexity of competent biomolecular logical gates and their effective utility towards diverse applications.

The transduction mechanism is employed to generate a signal output displaying the resulted state of the biorecognition events upon receiving the inputs. Typically, the outputs reflect the state alteration such as concentration increase/decrease of specific molecule (e.g., H_2_O_2_) and structural switch of identified biomolecules (e.g., DNA). These changes lead to the generation of physical signals in the luminescent, fluorescence, and electrical formats based on the designated constitution including incorporation of fluorescent probes, immobilization of bioreceptor, redox reaction of electrochemically active substances, and so forth. Once a threshold value, which well separates the inputs **0** and **1**, is appropriately defined, the “YES/NO” digital output is formed for conclusion. Besides this, in advanced technology, it is of particular interest that the computed output can be directed to actuation, which enables a further treatment. In connection with a stimuli-responsive interface, the output signal serves as a trigger to activate the desired reaction, which is able to, in a looped feedback module, relieve or improve the initial biochemical state characterized by the multiple inputs. Such a scheme allows the configuration of an intelligent “sense/act/treat” system.

## 3. Advances of Enzyme-Based Logical Gates for Sensing and Actuation

### 3.1. Sensing Based on Biocatalytic Reactions

Following the pioneering and conceptual works [6,7,8] validating a feasibility of digital sensors towards assessments of physiological and pathological conditions, Halámek *et al.* [9] reported a novel concept which harnessed parallel enzyme-based logical gates to yield an “injury code”, in which each code could be indicative of specified pathophysiological states prescribed by a truth table. Either **NAND** or **AND** gates were configured to assess the biomedical significance of soft-tissue injury (STI), traumatic brain injury (TBI), liver injury (LI), abdominal trauma (ABT), hemorrhagic shock (HS), and oxidative stress (OS), inputted with numerous biomarkers including creatine kinase, lactate dehydrogenase, norepinephrine, glutamate, alanine transaminase, lactate, glucose, glutathione disulfide, and glutathione reductase. By defining physiological and pathological levels of each biomarker as digital input **0** and **1**, binary outputs (*i.e*., **0** and **1**) were obtained via enzymatic processing of characteristic biocatalytic cascade reactions. An array of such the digital outputs multiplexed in a 6-bit injury code, enabling 64 (2^6^) unique injury combinations wherein each pathophysiological state was ascertained by a distinct “injury code”. The authors performed both electrochemical and optical techniques to evaluate individual enzymatic logical gates and have found satisfactory correlations between the two techniques, thereby confirming the validity of the transition of the experimental procedure from the optical to the electrochemical domain. While every injury was evaluated by using individual sensors in the traditional biosensing fashion, the success on this multiplex concept explored the merits of the logical gate architecture: conferring an inclusive injury code to assess multiple injury conditions.

Meanwhile, Zhou *et al.* [10] described the development of a rapid amperometric logic assay for screening patients suspected to have sustained traumatic brain injury (TBI) in a **NAND** gate manner. Compared with the TBI gate [9] with the two inputs of glutamate and norepinephrine, the authors selected glutamate (Glu) as one of the inputs based on its properties of being a freely-circulating metabolite and the most abundant excitatory neurotransmitter in the central nervous system (CNS). Moreover, lactate dehydrogenase (LDH) complemented glutamate as additional biomarker of broader diagnostic scope and thus was utilized as the other input. The utilization of LDH was referred to as a redundancy check on the assessment of a pathological state in an **AND** or **NAND** enzyme logical gate configuration to mitigate the occurrence of false positives. The biochemical cascade instigated by Glu and LDH relied on the first biocatalytic reaction (driven by glutamate oxidase) converting Glu to α-ketoglutaric acid (α-KG), which subsequently played the role of joint cofactor in the second biocatalytic reaction, driving l-alanine (ALA) to pyruvic acid (PYR) by alanine transaminase (ALT). Ultimately, PYR was oxidized to lactate (LAC) in connection with a depletion of NADH in the presence of the second input of LDH. The results revealed that, upon introducing pathological levels of both biomarkers that exceeded a pre-defined threshold taken from clinical data, the gate was able to trigger an immediate alert. All other combinations of the biomarker inputs corresponding to normal or anomalous physiological conditions did not cause the output of the logical gate to change state. In addition to the interference study (electroactive compounds such as ascorbic acid, uric acid, and acetaminophen) along with the resulting elevated background current and lesser difference between (**1**,**0**) and (**1**,**1**), most significantly, the authors evaluated the operation performance of the TBI logical system in serum. The biochemical complexity of the serum matrix was found to deteriorate detection of NADH, ascribed to an alteration of the detecting capability of the electrode. Proteins present in the serum may block the electrode surface. However, this problem can be resolved by elevating the initial NADH level to 600 μM, in which the differential current magnitude between the (**1**,**0**) and (**1**,**1**) can be increased to 26 nA, thereby yielding an unambiguous diagnosis of the pathological status. The authors concluded that the chemical constituents present in the serum had a sizeable effect on the output levels.

In light of the crucial effect of serum altering the operating excellence of digital sensors, Zhou *et al.* [11] biochemically investigated and optimized the logic analyses in biological fluid media, which was deemed to be a step closer towards practical biomedical applications. Aiming at attaining a reliable detection of injuries and making autonomous decisions towards timely therapeutic interventions, particularly in conditions in which a hospital treatment was not available (e.g., injuries in battlefield), clinically relevant biomarker characteristics of the selected injury models including liver injury (LI), soft tissue injury (STI), and abdominal trauma (ABT) were applied to test the integrity of the designed logical gates. In the route of aiming to understand the effect emanated from serum, the study of interfering substances in serum is of significance. The authors addressed the presence of pyruvate (approx. 40 μM in blood serum) since it could provide a false positive signal. In both LI and STI systems, the pyruvate production corresponded to the presence of one of the biomarker inputs. The high level of lactate as a common blood constituent was also underlined, given its conspicuously elevated concentration in the majority of traumatic injuries and its possible ability to prevent conversion of pyruvate to lactate in the enzymatic machinery. In addition, various ions in human serum such as Ca^2+^, Zn^2+^, Cu^2+^, Cl^−^, and PO_4_^3−^ can be potential inhibitors for the STI system. This thereby inspired the authors to add adequate Mg^2+^ and K^+^ ions to circumvent the interferent issues. Albeit there remain many technological challenges to make a human serum-based analysis a reality, the authors believe that the autonomous loop-based individualized integrated (sensing/release) medical systems will eventually have a substantial impact upon the treatment and survival of injured soldiers.

Distinct from the fact that most studies performed up to date have prepared artificial or biological fluids to study the logical gate system, Katz’s group, furthermore, presented the first result of a multi-analyte system activated by real samples from an animal model (liver injured pigs) [12]. While the analytical results in the artificially prepared fluid system reflected only the precision of the solution preparation, the patient-to-patient variations in the real biomedical analysis to alter the resulting distribution of the normal and pathological concentrations should be taken into account. Thus the authors utilized a Holcomb clamp technique to create two crush injuries in the liver parenchyma of male Yorkshire pigs weighting 15–20 kg. Control samples were also obtained using a similar procedure but without inducing liver injury to the animals. Serum samples from the injured animals (20 samples) and from a control group of animals without liver injury (35 samples) were collected for analysis. The concentrations of two enzymes, alanine transaminase (ALT) and lactate dehydrogenase (LDH), in the serum samples were utilized as inputs of an **AND** gate since they were known to be biomarkers characteristic to human liver injury, and the increased concentration of each of these enzymes separately could not be sufficiently specific to the diagnosis of liver injury. The normal physiological concentration of ALT and LDH at 0.02 and 0.15 U/mL^−1^, respectively, were employed as **0** of the inputs. Elevated levels of ALT (0.2 U/mL^−1^ and even to 2 U/mL^−1^) and LDH (1 U/mL^−1^) upon liver injury were referred to as **1**. By analyzing the absorbance change at 340 nm corresponding to the consumption of NADH upon the introduction of real samples into the logical gate system, the results allowed a statistically well-recognized difference, enabling a discrimination between the liver-injured and control pigs, and provided robustness of such **AND** gate-based analytical technique.

**Figure 1:**
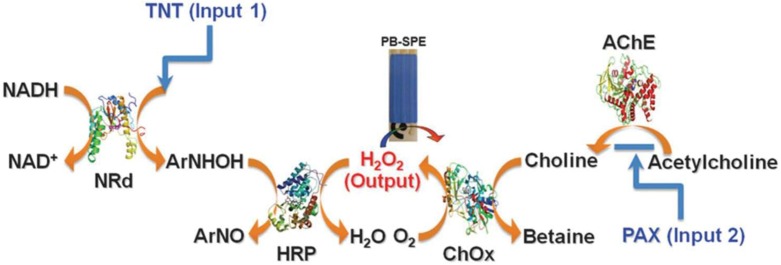
Biocatalytic cascade used to perform a NOR logic operation for the assessment of distinct classes of threat agents [13] (doi:10.1039/C0CC05716A). Reproduced with permission of The Royal Society of Chemistry.

Apart from the substantial body of endeavors along the route towards the demands for diverse injuries, the Wang and Katz groups [13] explored, for the first time, a unique biocatalytic cascade (a inherence of **NOR** gate) that was able to assess the presence of distinct classes of threat agents in a rapid and reliable manner, which was interesting and discriminated approach from conventional biosensors that commonly analyzed only one specific substrate. The capability of the new enzyme logic was studied employing the 2,4,6-trinitrotoluene (TNT) explosive and the paraoxon (PAX) nerve agent as the model inputs, as well as 2,4-dinitrotoluene (DNT) and methyl parathion (MPT) to demonstrate the detecting versatility of the enzyme logical gate towards a wide array of inputs (Figure 1). The logical gate leveraged a reaction cascade catalyzed by nitroreductase (NRd), horseradish peroxidase (HRP), acetylcholinesterase (AChE), and choline oxidase (ChOx) as backbone to process the TNT and PAX. Regarding TNT, H_2_O_2_ is partially depleted in the presence of the nitroaromatic explosive substrate through a NRd/HRP biocatalytic cascade. On the other hand, H_2_O_2_ is produced from the catalytic coupling of AChE/ChOx and acetylcholine. Cleverly, the authors enabled a unique feature of the cascade in which H_2_O_2_ levels could also be reduced through the inhibition of AChE by an organophosphate nerve agent. The concentration of H_2_O_2_ was recorded as an output by using a Prussian Blue (PB, a mediator)-modified screen-printed electrode. Ultimately, a decrease in the H_2_O_2_ level (and hence the current output) below a selected decision threshold was indicative of a “Hazardous” situation corresponding to the presence of an explosive and/or nerve agent, in line with the operation of a Boolean NOR gate. A reliable limit of detection of the system corresponded to 1.5 μg·mL^−^^1^ TNT and 1.25 μM PAX, estimated from iterated experiments until the [0 μg mL^−1^, 0 μM] level was separated by no more than six standard deviations from any non-zero level of the inputs.

### 3.2. Sensing and Actuation Processed by Enzyme Cascade Reactions

Based on the feature illustrated above, biocatalytic cascades that take the form of enzyme logical gates have represented an attractive route towards the development of rapid and high-fidelity analytical utilities applied to the determination of traumatic injuries. Advantageously, the generation of a single output from enzymatic logical gates makes the concept well-suited for incorporation with established controlled drug release units towards an “intelligent” interface with substantially prompted effectiveness of drug release. 

Indeed, enzymatic logical systems have been applied to regulate the state of signal-responsive materials such as a membrane and a nanoparticle to reversibly change the structure and material properties upon receiving signals from the biomolecular logical system [14,15,16]. However, only a few developed chemically switchable systems are controlled by biomedically meaningful biomarker substances, particularly at their physiologically relevant concentrations. Privman *et al*. reported the first example [17] of a switchable electrode interface logically controlled by the enzyme cascade processing biomarkers characteristic of liver injury. Alanine transaminase (ALT) and lactate dehydrogenase (LDH) were selected as the biomarker inputs for an AND logical gate with substrates (ketoglutarate and alanine) and cofactor (NADH). The resulting NAD^+^ was subsequently coupled with glucose dehydrogenase to generate gluconic acid, which acidified the solution. Such a pH alteration causes a reversibly structural change of P4VP (poly-(4-vinyl pyridine)), a polymer which exhibits a pKa of 4.7 and is bound to the electrode surface in a brush fashion. As the structure of the P4VP-modified electrode is dependent on the protonation state of the polymer, which is swollen and permeable for anionic redox species in its positively charged protonated state and remains in a shrunken (nonpermeable) state in neutral solution, the presence of binary inputted enzymes causes a permeability switch that allows an ON/OFF control of electrochemical activity of ferrocyanide. However, it should be noted that the P4VP membrane did not exhibit sufficient structural changes on the level of elevation of the biomedical inputs (*i.e*., ALT and LDH) since the pH value produced by the biocatalytic system did not reach a low enough value necessary for the restructuring of polymer protonation. The authors suggest that only severe injury conditions can provide changes to trigger the polymer opening and electrode activation.

Focussing on the intelligent drug-releasing interface of the same liver injury, the Katz group proposed another signal-processing biocomputing system by replacing the drug-releasing material [18]. The authors prepared a Fe^3+^-crosslinked alginate/poly-L-lysine/alginate (APA) microsphere, by layer-by-layer (LbL) deposition, to load rhodamine 6G dye as a mimic drug. As the Fe^3+^ cation formed a complex with citrate (binding constant at log *K* = 11.85), a ligand exchange reaction between citrate and alginate in the Fe^3+^-alginate complex initiated the alginated microsphere dissolution. Thus the authors demonstrated a citrate-activated microsphere dissolution wherein citrate was generated from a Boolean **AND** gate-configured biocatalytic cascade reaction leveraging alanine transaminase (ALT) and aspartate transaminase (AST) as biomarker inputs towards diagnosing liver injury. With glutamate and pyruvate as substrates for the ALT and AST joint biocatalytic cascade reaction, oxaloacetate was produced and subsequently converted to citrate with a further coupling of the third enzyme, citrate synthase. Model solutions containing ALT and AST at concentrations of 0.02 (logic input **0**) or 2 U·mL^−1^ (logic input **1**), characteristic of physiological and pathological scenarios, were applied to stude the gate machinery *in vitro*. By exerting a kinetic fluorescence measurement upon treatment of the APA microsphere with a solution containing various combinations of inputs, the input combinations (**0**,**0**), (**0**,**1**), and (**1**,**0**) resulted in a very slow and small increase of the fluorescence, thus proving that the protective two-layer shell on the APA microsphere kept the dye-loaded core intact. On the other hand, the input combination (**1**,**1**) exhibited an obvious increase of the fluorescence after the lag-period of ca. 40 min. The effective discrimination between the (**1**,**1**) input, indicative of the liver injury conditions, and all the other input combinations, corresponding to the normal physiology (**0**,**0**) or to another pathophysiology different from the liver injury (**0**,**1** and **1**,**0**), rationalized the feasibility of a drug-released activity processed by a biocomputing system.

Another example of the “**OR**” gate form instigated by two enzyme inputs was also reported by Radhakrishnan *et al*. [19]. A hollow capsule fabricated with protamine (PR) and chondroitin sulfate (CS) could be disintegrated in the presence of either trypsin or hyaluronidase (the enzymes over-expressed under conditions of cancer and inflammation). Since PR and CS are both FDA approved drugs, a destruction of the capsule triggers a drug-release system. As hyaluronidase (one of inputs in the gate machinery) can cleave N-acetylhexosaminic bonds between 2-acetoamido-2-deoxy-b-D-glucose and D-glucuronate in CS and trypsin acts with protamine, this system operates like a molecular **OR** logical gate, indicating that the presence of either input stimulus results in an output at **1** (namely the release of the encapsulated drug). The enzymatically inputted triggering of disintegration of the microcapsules has been confirmed by taking a fluorescence profile, using Confocal Laser Scanning Microscopy, of the capsules before and after treatment with the respective enzyme stimuli.

**Figure 2:**
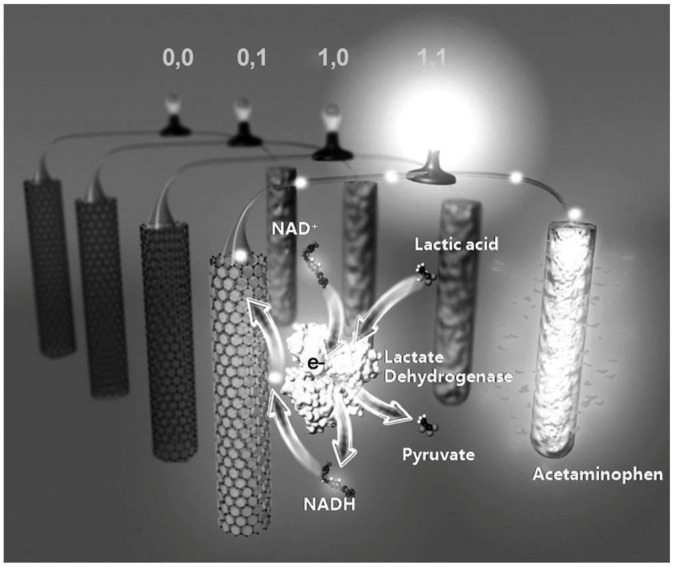
Illustrative diagram of the self-powered, biocomputing, logically-controlled “sense-act-treat” system based on a biofuel cell. Reprinted from [20] with permission of John Wiley and Sons. Copyright © 2012 WILEY-VCH Verlag GmbH & Co. KGaA, Weinheim.

In a more advanced development, the Wang group has described a self-powered, logically-controlled, integrated “sense-act-treat” system that is based on biofuel cells (BFCs) [20]. The system harnessed an enzyme-based, logically-controlled anode (a glassy carbon electrode modified with carbon nanotubes/Meldola’s blue (CNT–MDB/GC electrode)) and a drug-loaded, conducting polymer-modified cathode ((poly(3,4-ethylenedioxythiophene)/acetaminophen (PEDOT/APAP)-functionalized gold electrode) (Figure 2). To validate the concept, the system was operated in a potassium phosphate buffer solution containing nicotinamide adenine dinucleotide (NAD^+^) as a cofactor. Two biomarker inputs, lactic acid (LAC) and lactate dehydrogenase (LDH), were selected to an **AND** logic implementation corresponding to the selected model injury of abdominal trauma (ABT). The **AND** logic represented the situation in output **1** only when both LAC and LDH were present (input (**1**,**1**)). In this “abnormal” scenario, the LDH-catalyzed enzymatic reaction of NAD^+^ and LAC proceeded, which resulted in the formation of NADH and pyruvate; in the meanwhile, the open-circuit potential for the oxidation of NADH substantially decreased from 0.18 V to −0.10 V. The substantial swing in the open-circuit potential activated the oxidation of NADH and led to the concomitant reduction of PEDOT at the cathode. By virtue of the unique redox and doping/undoping properties of PEDOT to offer a structural change in meso/nanoporous manners, the entrapped APAP was subsequently released upon the reduction of PEDOT. Moreover, different combinations of the input signals, (**0**,**0**), (**0**,**1**), and (**1**,**0**) did not alter the open-circuit potential of the cathode. Thus, the PEDOT-APAP-modified electrode was suitable to serve as the cathode within the self-powered, controlled-release BFC. This BFC-based system has offered a biocomputing-based detection in connection with controlled drug-release functionality without the need of an external power source, which could serve as the core component of a closed-loop, self-powered autonomous medical diagnostic and drug-delivery system.

## 4. Advances of Nucleic Acid-Based Logic Gates for Sensing and Actuation

### 4.1. Sensing Based on DNA Processing

**Figure 3:**
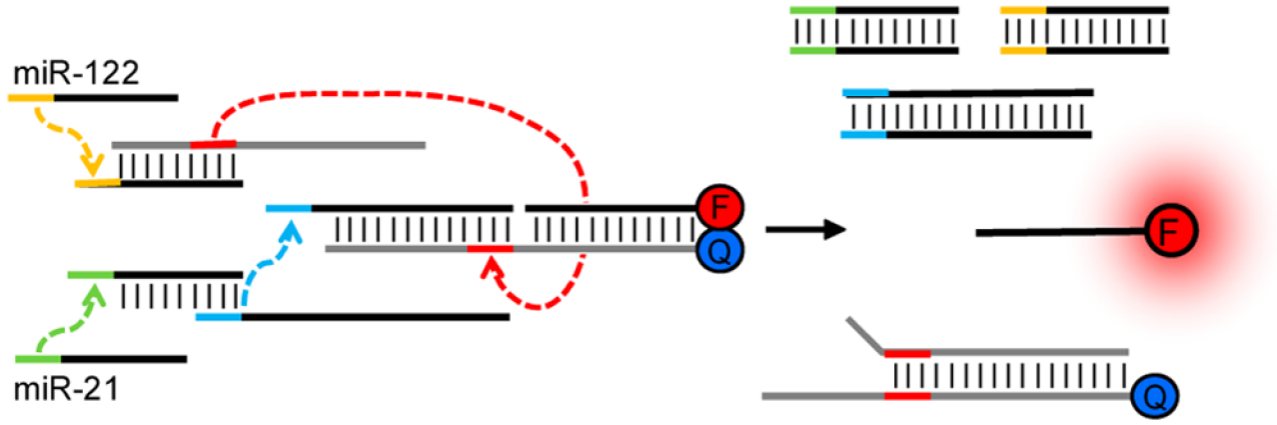
Schematic diagram of a translator AND gate incorporating miR-21 and miR-122 as inputs with resulting fluorescent output. Reprinted with permission from [27]. Copyright © 2013, American Chemical Society.

One of the fundamental mechanisms comprising DNA-based logical gates is the formation of Waston-Crick base-pairing [21,22] between complementary nucleic acids. This hybridization event is sequence-specific, kinetically rapid, and thermodynamically predictable, having been employed as a powerful principle in a myriad of molecular methods in past decades. Recently, a powerful approach, toehold mediated strand exchange, has been proposed for the accurate control of reaction kinetics involving multiple DNA strands [23,24], improving the understanding of the biophysics of nucleic acid hybridization and replacement. The formed hydrogen bonds between complementary strands are reversible, programmable, and can be readily controlled by inputted sequences, enabling facile design for logical operations [25,26]. An excellent example of executing simple DNA computation, based on toehold mediated strand displacement, in the biological environment (mammalian cells) towards diagnosis, was described by Hemphill and Deiters [27]. As depicted in Figure 3, the presence of two cancer biomarkers, microRNA miR-21 and miR-122, could be identified simultaneously in mammalian cells by the designed **AND** logic circuit. The authors utilized translator gates in which one DNA strand could be replaced by target RNA and thereby initiate the following displacement cascades to release the fluorescent probe from being quenched. The successful demonstration of DNA logical operation in live cells provided a promising approach for cellular microRNA profiling, laying the groundwork for the development of molecular computation in biological applications.

**Figure 4:**
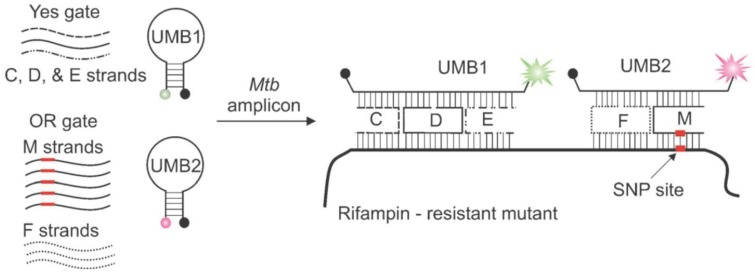
Integration of YES and OR logic gates based on the combination of two molecular beacons and DNA motifs for detection of Mtb and its resistance to Rif. Reproduced from [31] with permission of John Wiley and Sons. Copyright © 2012 WILEY-VCH Verlag GmbH & Co. KGaA, Weinheim.

In addition to simple complementarity between two strands, secondary structures of nucleic acids, such as DNA hairpins, three-way junctions, four-way junctions (Holiday junction), DX tiles [28,29,30], have been extensively harnessed for building logical gates and recognizing specific sequences. These structured probes and motifs, also constructed through intra- or intermolecular hydrogen bonds, exhibit great versatility towards logic sensors and intelligent devices by virtue of their thermodynamic stability and specificity [28]. The Kolpashchikov group wisely took advantage of the hairpin-structured probes combined with DNA motifs (DX tile) towards an efficient and cost-effective diagnostics to identify single mutations responsible for drug resistance in *Mycobacterium tuberculosis* (Mtb) [31]. They utilized a DNA-based **YES** gate to identify the presence of Mtb and an **OR** gate to analyze whether the Mtb DNA contain a mutation that confers rifampin (Rif) resistance (Figure 4). Specifically, the **YES** logical gate was composed of three DNA stands (C, D, and E) which formed two crossover points and a universal molecular beacon (UMB1). In the presence of the characteristic sequence of Mtb (either wild-type or Rif-resistant strain), the three strands formed a DX motif with the target DNA and UMB1. The **OR** gate, consisting of varied F and M strands, was designed to indicate the existence of any of the five drug resistance-conferring mutations within the hypervariable 81-nt region of the Mtb genome. In particular, each M strand was designated to recognize a specific mutant strain of Mtb resistant to Rif. In the presence of the cognate mutated analyte, corresponding F and M strands would form a fluorescent four-way junction with the second MB probe (UMB2) and the target sequence. This approach utilized a minimal number of fluorescent probes for the concurrent detection of Mtb and multiple mutations responsible for antibiotics resistance, successfully performing a complex diagnostic task by DNA computation with significantly reduced cost.

Besides the small, simple structures and motifs mentioned above, DNA molecules can also be harnessed to build various two- and three-dimensional architectures using so-called DNA origami method introduced by Rothemund [32]. DNA origami comprises single-stranded viral DNA and hundreds of short staple DNA strands. The addressability of DNA origami allows facile incorporation of multiple oligonucleotides, proteins, labels for biosensors, and so forth, on desired surface positions of the structure. In a technical note reported by Wang *et al.* [33], logical gates were designed on DNA origami for diagnosis of a disease by analyzing two indicators of heart failure, microRNA-21 (miR-21) and microRNA-195 (miR-195), as inputs. In their system, the **AND** logic computational module was implemented by two “Gate DNAs” and a biotinylated “signal DNA” assembled on capture probes at the surface of DNA origami. The two target miRNAs would hybridize with corresponding Gate DNA strands sequentially through toehold-mediated strand displacement, releasing the signal DNA. In the output module, there were three biotin-labeled DNA strands and two capture probes (bound by blocker DNAs) on DNA origami at designated positions which would present the output as “+” and “−” for the positive and negative result, respectively. Through the toehold region designed in the capture probes, the freed signal DNA could displace blockers and hybridize with the capture DNAs. After binding with streptavidin, biotins on the signal probe, together with the three immobilized biotinylated strands in the output module, a positive output “+” could be observed by atomic force microscopy (AFM). On the other hand, in the absence of miR-21 or miR-195, capture DNAs would remain blocked and the output would appear to be “−”. This system demonstrated a prototype of “lab on DNA Origami”, which is promising to the development of DNA origami-based smart devices for medical diagnostics and therapeutics.

**Figure 5:**
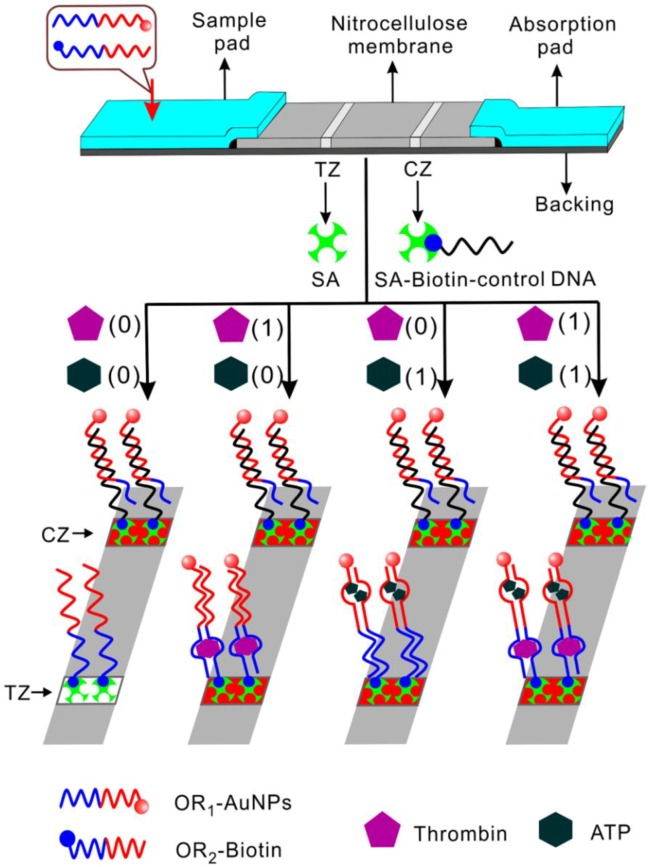
The strip “OR” logical gate using thrombin and ATP as inputs. SA: streptavidin, TZ: test zone, CZ: control zone. Reprinted with permission from [39]. Copyright © 2012, American Chemical Society.

Aside from the analysis of genetic sequences and microRNA, DNA molecules have been extensively exploited for the recognition of small molecules and proteins. These nucleics are aptamers, which have been discovered to recognize a wide range of ligands through a process called “Systematic Evolution of Ligands by EXponential enrichment (SELEX)” [34,35,36]. It has also been verified that some aptamers can be split into two fragments without significant deterioration in their ligand-binding functionalities [37,38]. Chen *et al.* [39] devised a strip logical system for proteins and small molecules based on target-induced assembly of split aptamers. In their study, two-analyte (thrombin and ATP) “**OR**” and “**AND**” logical gates were illustrated. There were two zones on the strip, test and control zones. In the **OR** gate (Figure 5), single stranded capture probes (OR_2_-biotin) consisting of one part of split aptamers for thrombin and ATP were immobilized on the test zone. The other part of the split fragments were coded by the signal probe (OR_1_-AuNPs) conjugated with a gold nanoparticle (AuNP). In the absence of both inputs, no signal probe was captured on the test zone, and thus no red color could be observed on the test zone, indicating a negative output. Upon the presence of either thrombin or ATP or both, the signal and capture DNA could together form integrated aptamers bound with their corresponding ligands. As a result, AuNP-conjugated signal probes would be captured on the test zone, and the accumulation of AuNPs could be visualized as a red band, reporting a positive output. The signal DNA with AuNP would also be captured by the immobilized control probe (SA-biotin-control DNA) in the control zone, forming a second red band. The authors further designed an **AND** gate using a similar strategy. In this case, the capture DNA in the test zone contained only one split part of thrombin aptamer, and the signal DNA labeled with AuNP comprised only a split fragment of ATP aptamer. In addition to the two strands, there is another recognition strand consisting of split parts of both thrombin and ATP aptamer. Only when both inputs existed, would a complex composed of both intact aptamers bound with their ligands be formed, capturing the AuNP-conjugated probe on the test zone and resulting in a visible red band. A red band would also appear on the control zone where signal DNA hybridized with the immobilized one. The assay was also demonstrated to exhibit high specificity and have the capability to detect target molecules in serum samples. This strip-based logic operation is simple, cost-effective, and can be developed into portable devices, providing a platform for point-of-care diagnostics.

It has long been elucidated that some metal ions interact with specific nucleotide bases to form DNA-metal base pairs [40]. Based on the stability and specificity of metal ion—DNA complexes, a variety of DNA logical gates have been reported, some of which were applied to analysis of the poison metals in water. Li *et al.* [41] demonstrated the construction of logical gates on the basis of the property that thymine–Hg^2+^–thymine (T–Hg^2+^–T) and cytosine–Ag^+^–cytosine (C–Ag^+^–C) coordination chemistry stabilized hybridization of oligonucleotides with T–T or C–C mismatches, respectively. In the **OR** logic design, T-rich and C-rich single-stranded DNAs, which were all labeled with a ruthenium (II) complex at one end, were fabricated on an electrode. In the presence of either or both ions (Hg^2+^ and Ag^+^), DNA probes would fold into a hairpin structure due to the coordination of mercury and silver ions with T–T and C–C couple, respectively. Therefore, the ruthenium (II) moiety would be confined near the surface, allowing the generation of electrochemiluminescent (ECL) signals. On the other hand, in the absence of any inputs, the ruthenium (II) molecule would stay away from the electrode due to the flexibility of single-stranded DNA; therefore, a low ECL signal was obtained. Based on the interaction of metal ions and specific nucleotides, the authors also designed several logical gates, such as **AND**, **NAND**, **INHIBIT**, and **NOR** gates, by utilizing varied arrangements and structures of T- and C-rich DNAs. Taking advantage of a similar principle, Zhang *et al.* [42] also developed “**OR**” and “**AND**” logical gates in a solution system, combining the common double helix-chelating dye, SYBR green, for signal output. Other examples reported by Ma *et al.* [43,44] have also demonstrated “**OR**” logic gate for the simultaneous determinations of hydrogen and potassium, as well as silver and hydrogen ions using a G-quadruplex-based, label-free, switch-on fluorescence detection method. In these studies, the designed “**OR**” logic operation was potentially applied to the detection of water sample in real life, such as river and drinking water, suggesting the practicability of DNA logical gates for analysis of metal ions.

**Figure 6:**
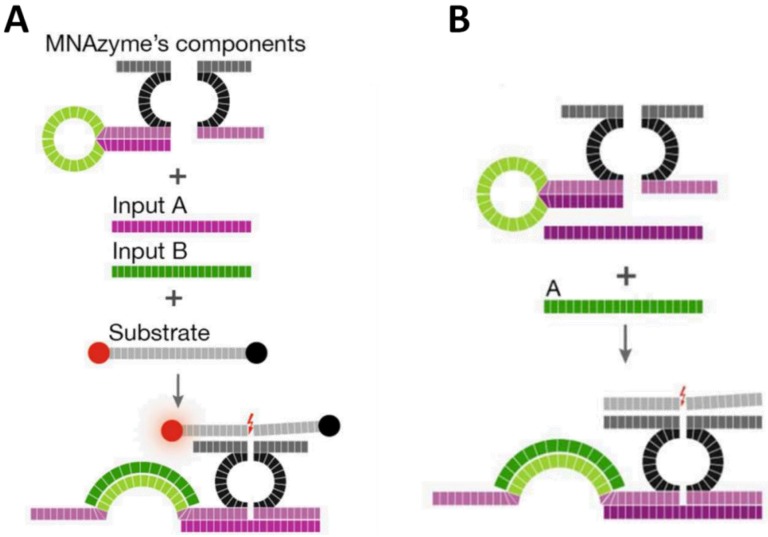
Schematic illustration of DNAzyme-involved (**A**) AND and (**B**) ANDNOT gate operations upon presence of their inputs. Reprinted with permission from [50]. Copyright © 2013, Rights Managed by Nature Publishing Group.

Apart from its famous form of double helix and ability to recognize nucleic acids, proteins, and small molecules, DNA can also be utilized as a catalyst. DNAzymes are deoxyribonucleic acid sequences that catalyze chemical reactions [45], among which the cleavage of RNA substrates is the activity most widely exploited in development of biosensors and therapeutics [46,47]. Like aptamers, DNAzymes also have been demonstrated to remain functional when being split into two segments [48,49]. Kahan-Hanum *et al.* [50] described a programmable DNAzyme library composed of various Boolean logic gates, including **YES**, **AND**, **NOT**, **OR**, **NAND**, **ANDNOT**, **XOR**, **NOR** and 3-input-**AND** gate, using cellular miRNAs and mRNAs as inputs (Figure 6). These computational modules were designed based on the cleaving activity of integrated Dz13 DNAzyme [51], which was split and would be combined only in the presence of specific inputs. Considering clinical purposes, the authors analyzed complex RNA combination, (miR31 **AND** miR21) **OR** (miR31 **AND NOT** miR125b) **OR** (c-myc), in cell lysates to determine breast cancer. The OR logic operation was composed of two **YES** gate, in which functional Dz13 could be assembled to cleave the substrate only in the presence of target RNA. The substrate was labeled with a quencher and a fluorophore at its two ends, respectively, emitting a strong fluorescence signal after being cleaved. For the **AND** gate (Figure 6A), there was a stem-loop structure, responsive to one input, on one arm of the split DNAzyme. Only when both target sequences existed could functional Dz13 form to catalyze the cleavage of substrates. The **ANDNOT** (Figure 6B) module was implemented by integrating the logic design of **AND** and **NOT** gates where an additional strand (“anti-input” molecule) complementary to one of the input sequences was used to hybridize with one of the two analytes. Thus, a strong signal could be obtained only in the scenario of one specific RNA sequence existing and the other not. The results indicated that a positive output (fluorescence signal) could be acquired only in conditions which met the requirements defined for breast cancer, showing the feasibility of complex Boolean logic constructed based on split DNAzymes.

### 4.2. Sensing and Actuation Processed by DNA

On account of their versatility, DNA-based logical gates have also been implemented to develop intelligent “sense and act” systems. The predictable and programmable property of nucleic acids makes them favorable for development of target-inducible molecular transport/drug delivery system. In the study of Wen *et al.* [52], DNA strands were fabricated on mesoporous silica (MS) to control the release of loaded drug responsive to specific environmental inputs. They designed an “**AND**” gate where a single stranded DNA containing the adenosine aptamer sequence (a) was modified on the MS drug carrier and hybridized with another DNA strand (b) labeled with gold nanoparticles (AuNP), which capped the pores on MS. Strand b contained the aptamer sequence for the other input, cocaine, and there was an overhang region on this strand in addition to the hybridized part. In the presence of both inputs, the unhybridized region on Strand b would specifically bind to cocaine to form the aptamer-target complex, resulting in the dissociation of a small section of the double strand. The destabilization of the double helix allowed the strand to bind with its ligand, adenosine, leading to the release of the AuNP-modified strand and loaded molecules. On the other hand, with either or neither inputs present, the release of drugs in MS could not be initiated, *i.e.*, no actions were taken by the smart device. 

Recently, the Tan group reported a molecularly assembled “Nano-Claw” capable of recognizing the expression levels of multiple cellular markers and autonomously activating therapeutic operations [53]. Aptamers were utilized as building blocks for a molecularly assembled logic robot, called the “Nano-Claw”. They built “**AND**” logic robots using oligonucleotide backbones as the scaffold, several structure-switchable aptamer/cDNA duplexes as “capture toes”, and logic-gated double-stranded DNAs as the “effector toe”. The “capture toes” recognized target cell-surface markers and thereupon released the corresponding barcode oligonucleotides for activation of the “effector toe”. Consequently, the “effector toe” received these barcode oligonucleotides as logical inputs, autonomously generating a molecularly computed output as a diagnostic signal (*i.e*., fluorescence) or therapeutic action. The results demonstrated that the “Nano-Claws” could effectively target cells co-expressing three different receptors in a single reaction. Moreover, the generation of output could be adjusted to report different levels of cellular expression by tuning the length of sequences hybridized with aptamers in “capture toes”. The authors believed that this robot could be further applied to basic biomedical research, accurate diagnosis, and potent therapy.

**Figure 7:**
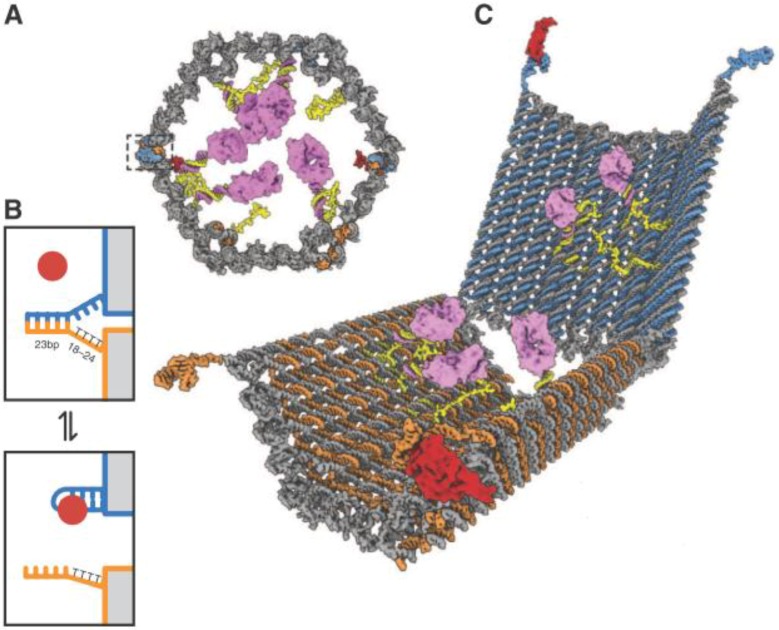
Design of aptamer-gated DNA nanorobot. (**A**) Schematic front view of closed nanorobot loaded with proteins. (**B**) The aptamer lock mechanism. (**C**) A nanorobot opened by ligand displacement of aptamer locks. From [54], reprinted with permission from AAAS.

In another design reported by the Church group, DNA was further utilized as a vessel carrying molecular payloads, having versatile functionalities including transport, sensing, computation, and acting [54]. By exploiting DNA origami technology, the authors created a three-dimentional nanorobot in the configuration of a hexagonal barrel (Figure 7). The barrel consisted of two domains that were associated in the rear by single-stranded hinges, and could be noncovalently fastened in the front by staples encoding DNA aptamer-based locks (boxed in Figure 7A) responsive to protein inputs. Two sets of aptamer-complement duplexes were incorporated on the left and right sides of the front of the barrel for sensing the targets, allowing an “**AND**” logic operation. The duplexes were composed of the aptamer strands anchored on one domain (blue strand in Figure 7B) and sequences partially complementary to the aptamers on the other domain (orange strand in Figure 7B). When both aptamers bound to their targets (red circle in Figure 7B), the lock duplexes would dissociate and result in a drastic conformational change of the nanorobot to expose the payloads on previously hidden surfaces inside the vessel (Figure 7C). The authors verified the feasibility and universality of the logic system by designing six different barrels with varied combinations of aptamer locks for simultaneous recognition of cellular surface markers. Moreover, they investigated the ability of the nanorobot to interfere with and modulate the intracellular signal transduction by specifically exposing the carried molecules. Their results demonstrated that the reconfiguration of the robot could only be induced by specific combinations of inputs, enabling modulation of signaling in targeted cells. Stemming from this concept, manifold logical gated nanorobots were further created by Douglas *et al.* [55], successfully performing complex computations in a living animal. In addition to gating the robots with protein cues, the authors designed several nucleic acid-controlled switches, which could be activated or negated by DNA payloads mounted in regulatory DNA robots. They utilized the system to construct architectures that emulate varied logical gates (**AND**, **OR**, **XOR**, **NAND**, **NOT**, **CNOT** and a half adder). As a result, the DNA origami robots in living cockroaches (*Blaberus discoidalis*) successfully modulated molecules that targeted their cells, providing a new type of biological computing platform particularly promising for retrieve of biological information and control of therapeutic molecules.

## 5. Advanced Biomolecular Logic Gates Operated with Enzyme/Nucleic Acid Integrated System

In this enzyme/DNA-integrated section, processing components of the biomolecular logical gate system usually are made up of both protein/enzyme and nucleic acid. The molecular recognition (e.g., hybridization of complementary genetic sequence) and catalytic abilities (e.g., DNAzyme) of DNA and RNA provided versatile sensing configurations compared to other molecular logical devices. Additionally, the enzyme offered the capability of signal amplification, which thereby could enhance the sensitivity of the biomolecular logical gate system.

**Figure 8:**
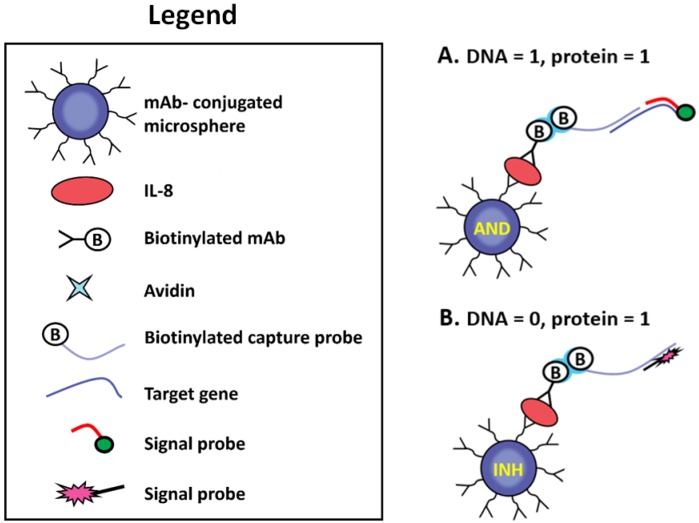
Schematic detection of (**A**) both a protein and a nucleic acid sequence as well as (**B**) only a protein. Reprinted with permission from [56]. Copyright © 2009, American Chemical Society.

A pioneering work which demonstrated both **AND** and **INHIBIT** (NOTIF) logical gates in response to the presence of both protein and DNA in a sample towards intelligent medical diagnostics was reported in 2009 [56]. The system was designed to determine whether both a protein and a nucleic acid sequence or only a protein is present in a sample, in which the results are interpreted by fluorescence via simple logical operation inherent with multiple molecular recognitions (Figure 8). As chronic obstructive pulmonary disease (COPD) or bronchial asthma is frequently associated with bacterial respiratory infections and is accompanied by the secretion of proinflammatory cytokines, such as IL-8 protein, a Boolean operation can be useful to describe the resulting logic of bacterial DNA and IL-8 protein. To this end, Cy3- and Cy5-tagged reporter probes were designed to complementarily hybridize to the bacterial DNA and a biotin-labeled probe, respectively. AND logic was represented by the situation where both inputs were present with a resulting “light on” of Cy3. Conversely, if only IL-8 protein was present in the sample solution without the existence of target DNA, only the Cy5-labeled signal probes hybridized to the biotin-labeled probe, which has been in complex with the second antibody against IL-8 protein. This is illustrated the INH logical gate, which switched on the Cy5 fluorescence emission solely in the (**0**,**1**) combination (where **0** and **1** were indicative of DNA and IL-8 protein, respectively). The authors integrated the logical-based sensing scheme into the fiber-optic microarray and claimed a potential application to direct screening of various medical conditions that are dependent on combinations of diagnostic markers.

Another example of the limited number of works, which include inputs in both protein and nucleic acid fashions was illustrated by Liu *et al.* towards diagnostics of prostate cancer [57]. As prostate-specific antigen (PSA) potentially gives a false-positive indication in diagnosing prostate cancer, an additional biomarker of survivin mRNA, which is over-expressed in many malignancies but rarely detected in normal differentiated adult tissues, was incorporated as input to mitigate the possibility originating from non-cancerous prostate diseases such as prostatic hyperplasia and prostatitis. The author modified the first antibody (clone ML00P01) against human PSA onto a nano-structured gold electrode and prepared a conjugate containing the second PSA antibody (clone ML00P02) and survivin ASODN (a sequence complementary with surviving mRNA in cancer cells). When both PSA and survivin mRNA coexisted in the tested solution, the clone ML00P02/survivin ASODN conjugate interacted with the PSA, which was in complex with clone ML00P01 on the electrodes. Then survivin mRNA hybridized with survivin ASODN to form a dsDNA structure. Subsequently Co(phen)_3_^3+^, an intercalation agent, intercalated the duplex DNA to give an electrochemical output with its redox activity. In the sole existence of PSA, the duplex DNA did not form, thereby only few Co(phen)_3_^3+^ intercalated ssDNA with a resulting low output current. On the other hand, the presence of only survivin mRNA disabled the outputting generation as a result of the absence of PSA and, consequently, the clone ML00P02/survivin ASODN did not conjugate onto the electrode. Thus a **AND** gate logical operation was realized by regulating the level of PSA and survivin mRNA. Moreover, the authors examined the logical gate-based analytical system in culture media of LNCaP cells and SMMC-7721 cells, as well as human serum, suggesting that such a device could be applied in the analysis of real samples in a complicated environment.

Discriminated from the most logical gates harnessing binary inputs for analysis, we have developed bimolecular circuits with a computing functionality against triple inputs and applied this to the identification of genetically modified (GM) materials and bacteria exhibiting multi-drug resistance [58]. Given that the three inputs are concomitantly present in the specific strain, an affirmative output indicates digital (**1**,**1**,**1**) output, indicating either the existence of the identified GM material or multi-drug resistance. In light of the extensive application of genetically modified technology in crops in connection with deficient assays in rapid and reliable fashions for quantity evaluation, we demonstrated a bimolecular circuit with a computing functionality mimicking the generation process of an event-specific gene and its feasibility in the analysis of GMOs. As the compatibility of inter-species genes with Nature has not been evaluated systematically, the long-term effects of GM foodstuffs on human beings and the global ecology remain unpredictable. This novel approach involved a promoter, new coding, and species genes fragments from a GM organism as relevant inputs to the circuit of Boolean logic gates. The facile processing of this logic gate system relied on integration of three hybridization activities, two affinity recognition events, and concatenated biocatalytic reactions that led to a resulting output, displayed as the electrochemical transformation of oxidized TMB (TMB(ox)) to reduced TMB (TMB(red)). Hybridization of the immobilized capture probes and the promoter segment forms the backbone of the **Identity** gate. Concurrently, an **AND** gate leveraging two hybridization events of the new coding and species genes, against FITC-conjugated and biotin-conjugated reporter probes, respectively, operated in parallel with the **Identity** gate. Subsequently, both glucose oxidase-labeled avidin (Av-GOx) and hydrogen peroxidase-labeled anti-FITC (α-FITC-HRP), bound to corresponding antigens, enabled an enzymatic cascade reaction to occur. That was, once the substrates (glucose and TMB(red)) were present, GOx catalyzed the oxidation of glucose to produce H_2_O_2_, which was further reduced by HRP along with the resulting TMB(ox) to configure the **AND** gate utility. Tthe biomolecular circuit-based system exhibited a high sensitivity that is reflected by the maximized gradient with respect to the GM percentage. Also, the system yielded the output current independent of ratio of genetically modified genes, which inferred that a giant body of non-GM DNA would not interfere with the assay. Previously, the authors have provided an alternative means for performing sophisticated multiplex analyses of selected GM events (A5547, NK603, T25, and LY038) in an array format, confirming that output **1** derived from the input combination (**1**,**1**,**1**) is accurately indicative of the presence of specific GM events.

**Figure 9:**
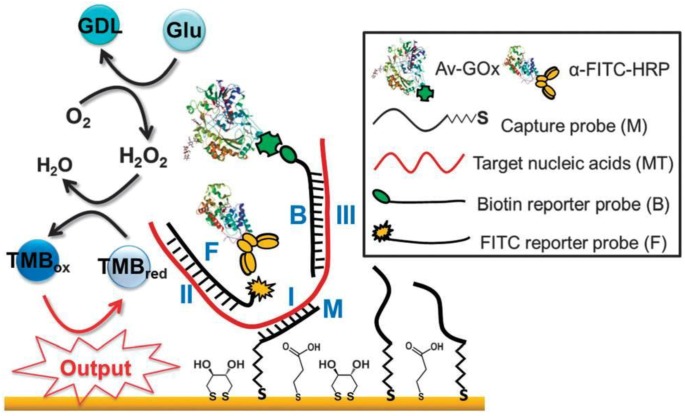
Biomolecular logical gate strategy for the detection of NDM-1-specific nucleic acids and its drug-resistance activity. [59]—Reproduced by permission of The Royal Society of Chemistry (doi:10.1039/c4cc01108b).

Motivated by a need to monitor the emerging bacteria exhibiting multi-drug resistance (MDR), we also reported a novel approach to identify the NDM-1-encoding gene (blaNDM-1) and concurrently to screen, by a tailor-designed biomolecular logical gate, two genetic fragments encoding the active sites bound to carbapenem [59]. The issue of MDR has recently been addressed after the first identification of metallo-*β*-lactamase-1 (NDM-1) in a *Klebsiella pneumoniae* isolate [60]. NDM-1 hydrolyzes the amide bond of *β*-lactam, which leads to nearly complete resistance to all *β*-lactam antibiotics of the bacteria. This brings a particular threat to public health as *β*-lactam antibiotics are clinically used as the last line of defense against MDR Gram-negative bacteria. Two oligo-peptides residing in the proximity of carbapenem (spherical zone with a radius of 1.5-fold that of the molecular diameter of carbapenem) were identified and reverse-translated for genetic fragments characteristic to the active site. The biomolecular logical gate composed of a hybridization of the NDM-1-specific fragment with the assembled capture probe as well as two hybridization events of the targeted segments (characteristic of the active site) against FITC-conjugated and biotin-conjugated reporters (Figure 9). The hybridization events were subsequently signaled by an enzyme cascade reaction coupling glucose oxidase and hydrogen peroxidase. The electrochemically signaling output was ultimately reflected by a cathodic reaction of oxidized 3,3*'*,5,5*'*-tetramethylbenzidine, TMB(ox). The results revealed that the new geometry of recognition proposed in this work was proved to greatly enhance the utility, compared to the conventional sandwich architecture, for analysis of long and highly structured nucleic acids. Moreover this design also exhibited high specificity and differentiation between mutated (Q123D and N220A) and wild-type DNA, accurately reflecting the potential loss of drug resistance in NDM and its host bacteria.

In addition, the Wang group described the first example of the ability to control the power output generated by a biofuel cells using DNAzyme-based biochemical signals processed in accordance with a **INH** logic operations [61]. However, this proof-of-concept study did not explicitly indicate the scope of ultimate application in connection with two inputs of adenosine deaminase (ADA) and adenosine monophosphate (AMP); as a consequence, the present article does not depict this work in details herein.

## 6. Molecular Logic Gates Built with Unique Materials and Microorganisms

A myriad of materials other than nucleic acids and enzymes, including nanoparticles, organic molecules or polymers, and cells, have also been utilized for construction of versatile logical gates. Recently, Lien *et al.* reported functional logical gates based on the inducible peroxidase-like activities in gold nanoparticles (AuNPs) for identification of lead (Pb^2+^) and mercury (Hg^2+^) ions [62]. In the mechanism for detection of Pb^2+^, the coexistence of AuNPs and Pt^4+^ would lead to self-deposition of platinum on the AuNPs’ surface as a result of the strong aurophilic interactions between Pt^4+^ and surface Au atoms/ions. When S_2_O_3_^2−^ was presented, it would form complexes with Au^+^ on the Pt-AuNP surfaces, which then facilitated the deposition of lead on the nanoparticles. Consequently, the Pb/Pt-deposited AuNPs exhibited potent peroxidase-like activity. For the analysis of Hg^2+^, Bi^3+^-AuNPs were employed. The presence of Hg^2+^ would switch the catalytic activity of Bi^3+^-AuNPs from peroxidase-like to catalase-like, producing bubbles by turning H_2_O_2_ into H_2_O and gaseous O_2_. The authors testified the feasibility of the sensing strategy by determining concentrations of spiked Pb^2+^ and Hg^2+^ in tap, river, or lake water samples. Furthermore, combining the two reactions, the authors built an “**AND**” logic operation for simultaneous visual detection of lead and mercury ions, which would be favorable for real applications. 

**Figure 10:**
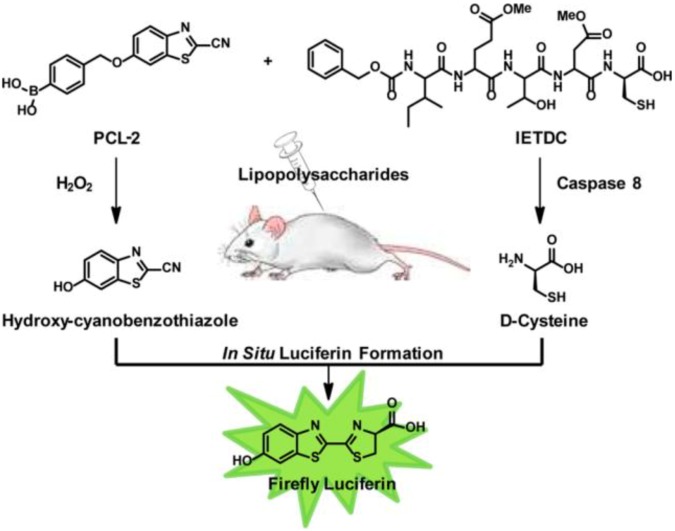
The design for simultaneous detection of H_2_O_2_ and caspase 8 activity through chemical reactions releasing Hydroxy-cyanobenzothiazole and D-cysteine and *in situ* formation of firefly luciferin. Reprinted with permission from [63]. Copyright © 2013, American Chemical Society.

In addition to nanoparticles, synthetic organic compounds are also excellent materials for creating computational modules based on their chemical reactivity towards specific biochemical species and special optical properties. For instance, the Chang group described an “**AND**” logic strategy (Figure 10) for dual-analyte detection in living animals [63]. They developed two organic molecules. One was Peroxy Caged Luciferin-2 (PCL-2), which would release 6-hydroxy-2-cyanobenzothiazole (HCBT) following the reaction with hydrogen peroxide. The other was a peptide-based probe, z-Ile-Glu-ThrAsp-D-Cys (IETDC), which could be hydrolyzed to produce D-cysteine by active caspase 8. The two released molecules HCBT and D-cysteine formed luciferin *in situ*, resulting in a bioluminescent signal if and only if both biochemical processes proceeded. By using the combination of the two organic probes, the authors successfully and selectively monitored the concomitant presence of H_2_O_2_ (reactive oxidative species, ROS) and active caspase 8 in living cells and animals to report the biological state of acute inflammation. This study offered a potential bio-imaging tool for *in-situ* monitoring of multiple analytes of physiological significance. In another communication published recently, Hettie *et al.* also described a three-input “AND” logic fluorescent molecular logical gate based on the coumarin-3-aldehyde scaffold for application in neuronal imaging [64]. This **AND** gate was responsive to glutamate, zinc, and pH through functional groups incorporated into the π-system of their designed fluorophore. The aldehyde at the coumarin 3-position reacted with glutamate to form imines, and thus created a binding pocket for coordination with zinc. Following the binding of the probe with glutamate and zinc, the hydroxyl group at the 7-position sensed the change in pH, turning on a fluorescence response. The authors believed that this sensor could serve as a prototype, which potentially offers significant insight into mechanisms of neuronal functions and diseases by imaging the co-release of neural transmitters.

**Figure 11:**
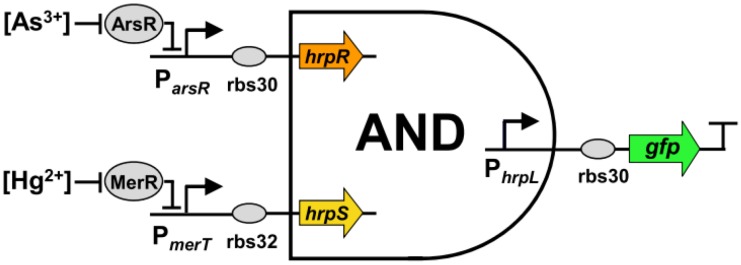
The **AND** logical cellular sensor sensitive to arsenic and mercury ions. Reproduced from [65] © 2012 Elsevier B.V. All rights reserved. Under the Creative Commons Attribution license (http://creativecommons.org/licenses/by/3.0/).

Besides inanimate materials, living organisms can also be manipulated to execute computational tasks. Wang *et al.* engineered varied cell-based logical sensors by utilizing the natural signaling pathways of the host cells, or by exploiting sensory functionalities from other bacteria [65]. In a cellular “**AND**” gate they constructed for simultaneous detection of arsenic (As^3+^) and mercury (Hg^2+^) ions (Figure 11), two heterologous genes, hrpR and hrpS, and one σ^54^-dependent promoter, hrpL [66,67], were integrated and connected to the output expression of a green fluorescent protein (GFP). The presence of As^3+^ and Hg^2+^ promoted the expression of hrpR and hrpS, respectively, to produce two enhancer-binding proteins that formed a heteromeric complex, which activated hrpL promoter, resulting in the generation of GFP. On the basis of this principle, the programmable and scalable cellular sensing system could be rewired towards different input combinations to drive biochemical outputs, facilitating quantitative analysis of environmental contaminants or disease-related signals.

Some logical gates built by utilizing polymers and nanoparticles also have been reported to be capable of acting on the sensed inputs, such as bioresponsive drug carriers. For instance, the Almutairi group synthesized a novel polythioether ketal nanoparticle [68] in which the thioether moiety acts as a reactive oxygen species (ROS)-sensitive switch that increases hydrophilicity of the particle when it is oxidized from a thioether into a sulfone. This reaction allows rapid acid-catalyzed degradation of the ketal groups in the polymer backbone at pH 5–6 [69,70,71] and consequently results in the release of payloads. Therefore, this nanoparticle functions as an “**AND**” logic circuit for sensing both ROS and low pH which are typical of inflamed tissue, accordingly acting on the diseased site. Besides, logically-gated materials may also enable co-delivery of multiple drugs. In a study conducted by Zhou *et al.* [72], DNA-gated mesoporous silica nanoparticles (MSN) functionalized with disulfide-linked acridinamine (a DNA intercalator, also an antibacterial and antitumoral drug) were designed to control the release of two loaded drugs. In the presence of dual stimuli (glutathione (GSH) and DNase I), the first drug encapsulated in MSN could be released due to the digestion of capped DNA duplexes by DNase I, and the second drug was set free after the reduction of disulfide bonds by GSH. The author believed that the smart nanodevice, which functioned as an “**AND**” logical gate, could be precisely controlled in complex situations and held potential to improve therapeutic efficacy at the target sites. 

## 7. Conclusions and Perspectives

In this review, we have highlighted the recent advances in the development of biomolecular logical gates-based biosensors and actuators. Biomolecules function as the fundamental operating elements of complex multi-input processing which enables molecular recognition, signal transduction, and actuation. Despite the fact that a substantial body of achievement has been described in this review, the field in which the built-in logical gates are utilized as the core entity of biosensors remains in its infancy.

Certainly, the built-in molecular logical gates have intrinsically brought biosensors many advantages. For example, more efficient and effective configuration can be attained in which minimum effort is necessary prior to the application of an according intervention. A prominent paradigm is shown in Kolpashchikov’s work wherein an **identity** gate along with an **OR** gate were concerted to answer if the rifampin resistance was present. On the contrary, the most advanced commercial assay, Cepheid’s Expert MTB/RIF, which is performed using five expensive molecular beacon probes along with a five-channel fluorescent reader, is redundant and can be simplified by the utilization of nucleic acids-based logical gates [31]. The concept of logical gate-processed multiplex screening of multiple genetic fragments or point mutations serves as the core functionality of a smart platform that circumvents the need of cumbersome operations and high costs commonly involved in currently established strategies for multi-analytes. 

A further benefit is that the approach using logically-gated sensing devices is capable of offering a decisive answer on the basis of molecular computations to exclude potential errors caused by personnel. In connection with an appropriate transduction mechanism, for example, electrochemical devices with inherently desirable properties of simplicity and robustness, molecular computing can be promisingly employed for its mid-sized screening capability [58]. Such a configuration is particularly desirable in in-field measurements compared to the sophisticated platform employed in mass screening processes.

Nevertheless, a new strategy should be developed to overcome the challenges and limitations the current design of logical gates encompass. An important challenge is to increase the dynamic difference between the **0** and **1** outputs (namely a well-defined YES/NO answer). As for the ultimate usage in medical emergencies, it is frequently demanded to discriminate a relatively small difference between the physiological and pathological levels, for which it is challenging to generate a well-defined interpretation. Furthermore, it is desired that the output is ideally given in nonlinearity with sharp transitions between **0** and **1**. This indicates that extensive research efforts aiming at the optimization of the biomolecular reactions/activities or the design of a chemical “filter” system [73,74] are necessary. In addition, from a systematic point of view, while the numerous applications of the biomolecular logical gate system harnessed the simplest **AND**/**NAND** and **OR**/**NOR** logical gate, scaling up the complexity of the bioprocessing system from the simple logical gate to the circuited network is a particularly challenging task in this field of research. Although there are a few paradigms [58,59] revealing a simple logical circuit system to process triple inputs concurrently, they remain in exemplified utilizations effective to interpret specified diagnoses, and are inadequate to describe diverse applications with a large versatility.

To put things into perspective, to fuel the further development in this valuable field, the application of the logically gate-based strategy to resolve significant biomedical tasks is required. This underlines the importance of the Boolean circuit built-in sensing scheme, which leverages a contingent of logical gates to process diverse analytes. Upon entrance of input information, the processing through the contingent of logical gates, configured as either parallel or serial linkages, would be akin to the operation of a “decision tree”. Genuinely expediting the operation of a “decision tree” on the basis of biomoleular logical gates offers the opportunity of the decision excluding potential errors. As nucleic acids have evolved as profitable utility for the next generation of information processing by virtue of their advantageous characteristics including reversible hybridization, rich structural complexity, and biocatalytics, they are promising candidates for the main element constituting the biomolecular circuit with given functionalization as the decision tree. Ultimately, one of the goals in developing biomolecular logic-based biosensors is to achieve the detection *in* *vivo*. Thus it might be worthwhile to further address the biofuel cell powered biosensor, such as found in the work reported by the Wang group [20]. Upon coupling the self-powered biosensor with a well-designed and fabricated signal-responsive interface, an intelligent, closed-loop, and *in* *vivo* “sense-act-treat” biological system can be offered. Diverse and significant fields would benefit from the resulting digital system, including assorted monitoring in human life that requires immediate intervention or proper action on the basis of reliable analytical data.
